# QUIN 2.0 - new release of the QUaternary fault strain INdicators database from the Southern Apennines of Italy

**DOI:** 10.1038/s41597-024-03008-6

**Published:** 2024-02-12

**Authors:** Giusy Lavecchia, Simone Bello, Carlo Andrenacci, Daniele Cirillo, Federico Pietrolungo, Donato Talone, Federica Ferrarini, Rita de Nardis, Paolo Galli, Joanna Faure Walker, Claudia Sgambato, Marco Menichetti, Carmelo Monaco, Salvatore Gambino, Giorgio De Guidi, Giovanni Barreca, Francesco Carnemolla, Fabio Brighenti, Salvatore Giuffrida, Claudia Pirrotta, Filippo Carboni, Luigi Ferranti, Luisa Valoroso, Giovanni Toscani, Massimiliano R. Barchi, Gerald Roberts, Francesco Brozzetti

**Affiliations:** 1 DiSPuTer, Università degli Studi “G. d’Annunzio” Chieti-Pescara, Chieti, Italy; 2 CRUST - Centro inteRUniversitario per l’analisi Sismotettonica Tridimensionale, Chieti, Italy; 3 CNR, Istituto di Geologia Ambientale e Geoingegneria, Monterotondo 00016 Rome, Italy; 4 Dipartimento della Protezione Civile, 00193 Rome, Italy; 5 Istituto di Geologia Ambientale e Geoingegneria (IGAG) del Consiglio Nazionale delle Ricerche (CNR), Monterotondo 00016, Rome, Italy; 6 Institute for Risk and Disaster Reduction, University College London, London, UK; 7 Department of Earth and Planetary Sciences, Birkbeck, University of London, London, UK; 8 Università degli Studi di Urbino Carlo Bo, Urbino, Italy; 9 Dipartimento di Scienze Biologiche Geologiche e Ambientali, Università di Catania, 95129 Catania, Italy; 10 Istituto Nazionale di Geofisica e Vulcanologia, Osservatorio Etneo-Sezione di Catania, Catania, Italy; 11 Institute of Earth and Environmental Sciences (Geology), Albert-Ludwigs-University Freiburg, Freiburg, Germany; 12 Dipartimento Fisica e Geologia, Università Degli Studi di Perugia, Perugia, Italy; 13 DiSTAR, Università degli Studi di Napoli Federico II, Naples, Italy; 14 Istituto Nazionale di Geofisica e Vulcanologia, Rome, Italy; 15 Dipartimento di Scienze della Terra e dell’Ambiente, Università di Pavia, Pavia, Italy

**Keywords:** Tectonics, Structural geology, Natural hazards, Geodynamics

## Abstract

QUIN database integrates and organizes structural-geological information from published and unpublished sources to constrain deformation in seismotectonic studies. The initial release, QUIN1.0, comprised 3,339 Fault Striation Pairs, mapped on 445 sites exposed along the Quaternary faults of central Italy. The present Data Descriptor introduces the QUIN 2.0 release, which includes 4,297 Fault Striation Pairs on 738 Structural Sites from southern Italy. The newly investigated faults span ~500 km along the Apennines chain, with strikes transitioning from ~SE to ~SW and comprehensively details Fault Striation Pairs’ location, attitude, kinematics, and deformation axes. Additionally, it offers a shapefile of the fault traces hosting the data. The QUIN 2.0 release offers a significant geographic extension to the QUIN 1.0, with comprehensive description of local geometric-kinematic complexities of the regional pattern. The QUIN data may be especially relevant for constraining intra-Apennine potential seismogenic deformation patterns, where earthquake data only offer scattered or incomplete information. QUIN’s data will support studies aimed at enhancing geological understanding, hazard assessment and comprehension of fault rupture propagation and barriers.

## Background & Summary

Open shared databases in seismic hazard research provide a landmark platform for scientists, researchers and policymakers to access and analyse vast geological and seismological data, enabling a deeper understanding of earthquake hazards and improving our ability to assess and mitigate seismic risks.

In a recent Data Descriptor^1^, QUIN (QUaternary fault strain INdicators) was introduced as a comprehensive geological database designed to integrate and organize geological information to analyse Quaternary deformation and strain, primarily for seismotectonic studies and geodynamic modeling. The initial release^1^ focused on the Northern and Central Apennines extensional belt and included structural field data on fault geometry and kinematics: specifically, 3,339 Fault Striation Pairs (hereinafter “FSPs”) mapped at 445 Structural Sites (hereinafter “SSs”) and derived strain indicators were included in the database. Given that the regional stress field in Italy has remained relatively unchanged over the last 3 million years^2–4^, it is feasible to extend the observation of structural data relevant to seismogenic purposes at least to the entire Quaternary period, encompassing the last 2.58 million years^5^. Covering the extension back this far opens up a wealth of data that can significantly improve our understanding of present deformation patterns which often appear incomplete when relying just on earthquake data.

Open-access databases on present-day stress patterns are widely available in the literature (*e.g*.^6–12^). However, they primarily rely on indicators such as focal mechanisms, borehole breakouts, and overcoring data. Less information is available on geological fault data, which might provide insights into stress variations at the local and regional fault scale (*e.g*.^13–21^). In this regard, Italy stands out as an exception. Indeed, due to the excellent exposure conditions of the intra-Apennine normal faults and their accessibility, some fault/slip databases are available in the literature (*e.g*.^22–26^), but they do not refer to southern Italy and do not provide detailed strain pattern data as those given in the QUIN database.

**Fig. 1:**
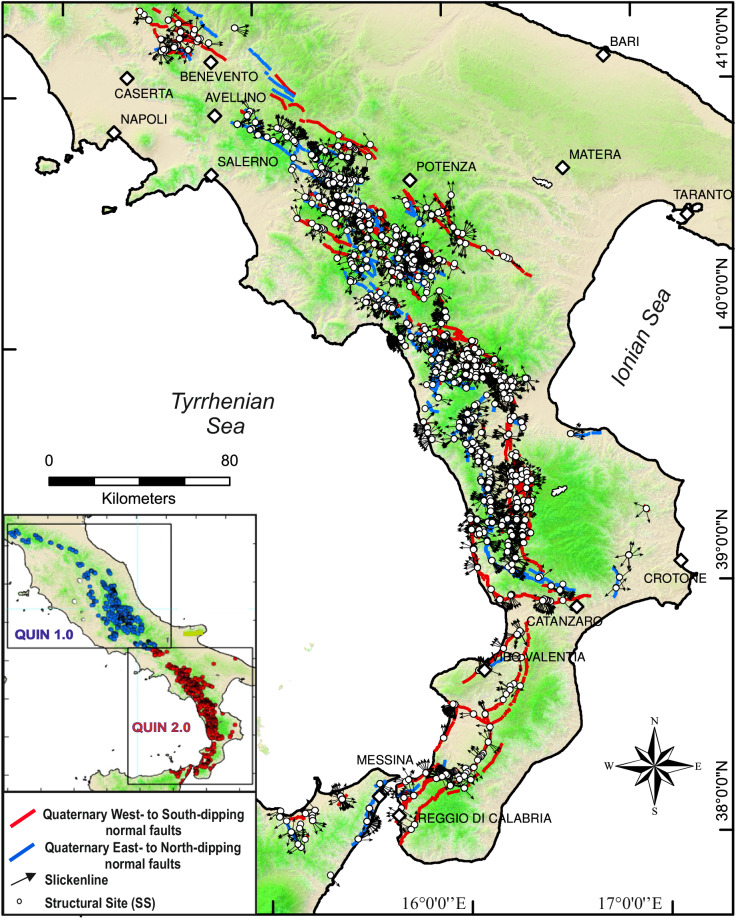
Map view of the QUIN 2.0 database projected on a shaded relief map of the Southern Apennines of Italy and northeastern Sicily along the trace of the faults (red and blue lines) hosting the QUIN’s data (*i.e*., HF). White circles represent the location of the 738 SS while arrows indicate the slip direction of each of the 4297 FSP. The inset shows the data coverage of the QUIN 1.0^1^ (Northern and Central Apennines) and the QUIN 2.0 releases (Southern Apennines and northeastern Sicily).

**Fig. 2:**
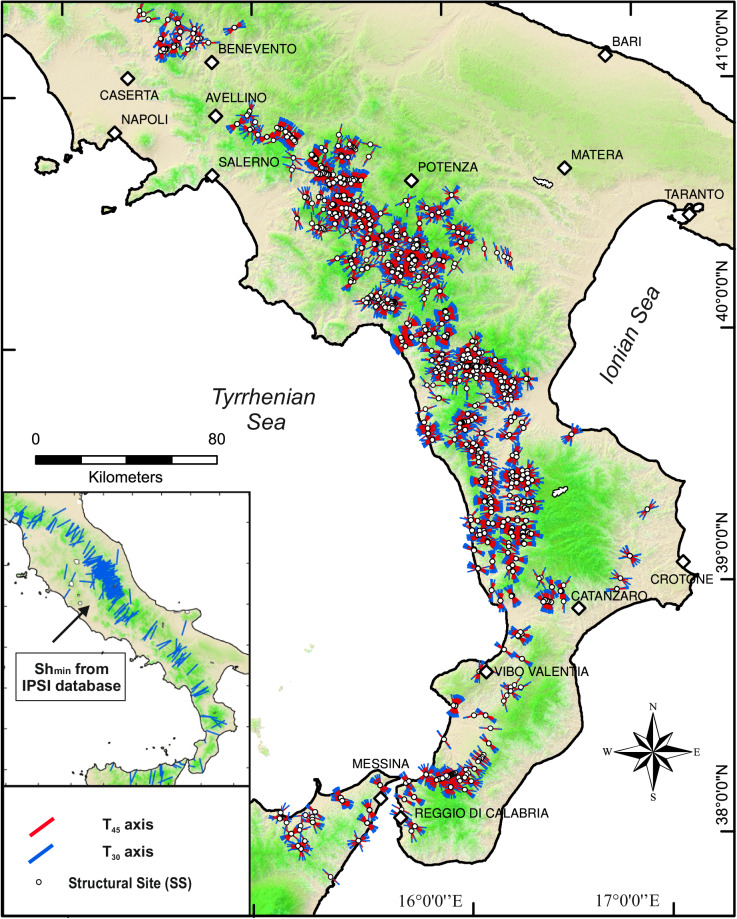
T_30_- and T_45_-axis computed in this database from the input fault/striation pairs data (see next section), on a shaded relief map of the Southern Apennines of Italy and northeastern Sicily. The inset shows the Sh_min_ orientation from the IPSI focal mechanism database^7,9^ for comparison with the QUIN database (see “Technical validation” section).

The methodology employed in QUIN and the criteria used to compile the database were fully described by Lavecchia *et al*.^1^, together with its main features and applications. Lavecchia *et al*.^1^ also included examples illustrating how the database can be used to investigate the entral Apennines region’ seismic potential and deformation patterns. We here present the QUIN 2.0 database which extends the coverage to the Quaternary extensional faults in the Southern Apennines of Italy and Sicily, along a length of ~500 km (Figs. 1, 2). QUIN 2.0 incorporates a wide collection of FSP records, including both unpublished (2006) and literature data (2291), distributed along 738 SS. For each FSP, the database provides information on the major Quaternary fault hosting the SS (Host Fault, hereinafter “HF”), geometric and kinematic parameters, and a quality assessment of the data. The additional FSP records of the QUIN 2.0 significantly enlarge the area considered by QUIN 1.0 (Fig. 1), providing a comprehensive view of the potential seismogenic structures of Central and Southern Italy.

The Southern Apennines area is characterized by an interconnected pattern of normal-to-oblique faults that generated some of the most destructive earthquakes in Italy^27^, such as the Campania-Lucania 1980 earthquake (M_w_ 6.9)^28^, the Messina Strait 1908 earthquake (M_w_ 7.1)^29–31^, the Basilicata 1857 earthquake (M_w_ 7.1)^32–35^, the Southern Calabria 1783 seismic sequence (M_w_ 7.1)^31,36,37^ and the March-June 1638 Central Calabria earthquakes (M_w_ 7.1)^38,39^. Despite this, in contrast to the Central and Northern Apennines^21,40–47^, since 1981 the area has undergone only minor-to-moderate seismicity (M_wmax_ 5.7^48^) with a consequent lack of extensive information on the active (instantaneous) and seismogenic stress field.

The long-term structural data provided by QUIN 2.0 advances the understanding of the Quaternary deformation patterns in the Southern Apennines of Italy and Sicily, providing valuable insights for seismotectonic studies and related research fields. The database can be integrated with geodetic^49^ and seismological data to carry out formal stress inversions at the regional scale. This is useful for investigating local-scale effects that control active deformation, seismogenic faulting, and seismic hazard in a region where high seismogenic potential is suggested by both historical seismicity and Quaternary faults, even if only moderate seismicity was recorded in instrumental times. Furthermore, the database can be exploited for further elaborations on structural analysis, high-detail fault segmentation and distribution (*e.g*.^50–53^), geodynamic reconstructions and seismic hazard as well as for geothermal and petroleum exploration.

By freely sharing and collaborating on multidisciplinary data and fully integrating structural and seismological data, the scientific community can foster innovation, accelerate seismotectonic research progress, and facilitate evidence-based decision-making, ultimately leading to more effective earthquake preparedness and resilience strategies.

## Methods

### QUIN 2.0 Database: structure and building process

The development of the QUIN 1.0 database^1^ involved a systematic approach, integrating existing data from the literature with newly acquired information. The database was aimed to create a comprehensive and detailed repository of Quaternary fault/slip data for the Northern-Central Italy intra-Apennine extensional belt. In this second release, focused on the extensional belt of southern peninsular Italy and north-eastern Sicily, we followed the same approach and method introduced for QUIN 1.0, which we briefly recall below. A complete description of the QUIN database’s structure, building process and usage, can be found in the first release^1^.

The QUIN database-building process consists of several key steps needed for the database’s comprehensiveness. First, we reviewed literature sources to gather relevant fault-related data. Second, we incorporated new and unpublished data from the present paper’s authors. Third, we carried out targeted campaigns to collect missing information and bridge data gaps in the database. These campaigns involved field surveys, geological mapping, and fault plane analysis. The acquired data were integrated into the database, ensuring a complete coverage of FSPs and SSs. The latter, in the QUIN database, are grouped into four distinct categories based on their proximity and characteristics concerning the HFs: (1) SS located on or near the main HF trace within its damage zone, (2) SS representing syn-kinematic faults outside the HF damage zone, (3) SS consisting of faults observed along structures antithetic to the HF, and (4) SS comprising scattered Quaternary fault planes, not directly related to any major HF. This categorization system facilitates efficient organization and retrieval of data. A quality control process was implemented to ensure data accuracy and reliability. Data validation and verification procedures were performed to identify and rectify possible inconsistencies or errors. Detailed information may be found in Lavecchia *et al*.^1^.

Starting from the FSPs data, we perform kinematic analysis and deformation axes calculation. After calculating the rake values (Aki and Richards^54^ convention), we classify the FSPs in kinematic classes corresponding to pitch ranges of 0–30° for strike-slip faults, 30–60° for oblique faults, and 60–90° for dip-slip faults (see Data Record section). Additionally, the attitudes of the principal deformation axes are determined based on two scenarios: a compressional axis at 30° or 45° in the slip plane. The first scenario rests on Anderson’s theory, which suggests an angle of ~30° between the rupture plane and the maximum principal stress (σ_1_) in the upper crust. The second scenario considers conventional P-T deformation axes at 45°, as obtained from focal mechanisms and classically used for an initial depiction of SH_min_ horizontal trajectories. The theory beneath the previous choices is discussed in Lavecchia *et al*.^1^. The above calculations and graphic representations are performed using the *F-SPA tool* specifically developed in MATLAB by Andrenacci *et al*.^55^ for use in QUIN.

### Host faults and their tectonic context

Following Lavecchia *et al*.^56^, an updated and detailed review of the HF traces was performed in the present Data Descriptor and a new fault traces map was carried out on a GIS platform (Fig. 1). The database is stored in ZENODO^57^ in shapefile format (.shp) and contains 245 HF records. The related attribute table provides information on the individual HF name and dip direction, as well as on the name and the age of activity (i.e., fault onset) of the fault system containing the HFs. Furthermore, in the attribute table we report information on the possible involvement of the fault in historical/instrumental earthquakes. The QUIN 2.0 HF database covers ~500 km of the Campania, Lucania, and Calabria extensional belt in Southern Italy, encompassing the Peloritani mountains in northeastern Sicily^58–61^. The fault traces were extracted from the literature and were integrated/updated with data from unpublished maps related to the newly acquired FSPs (i.e., collected in the new field campaigns). In particular, we consulted the online version of the Structural and Neotectonic Models of Italy (scale 1:500.000; https://www.socgeol.it/438/structural-model-of-italy-scale-1-500-000.html), the ITHACA catalog (https://sgi.isprambiente.it/ithaca/viewer/) and the sheets of the Geological Map of Italy at 1:100.00 (http://sgi.isprambiente.it/geologia100k/) and 1:50.000 (https://www.isprambiente.gov.it/Media/carg/) scales. Other detailed traces of Late Pleistocene-Holocene faults, accessible on online maps and repositories, together with all the published papers also used to compile the QUIN fault/slip database, were also considered^17,19,26,28,30,35,36,61–97^. We opted not to utilize the DISS database (https://diss.ingv.it/) for QUIN host faults, as it serves different applications and lacks the requisite details for our specific purposes. We derived the age of the fault-systems’ activity from the geological literature^3,28,35,38,62,63,65,98–109^ and from the evidence of earthquake activity in historical and instrumental times^27,110^.

**Fig. 3:**
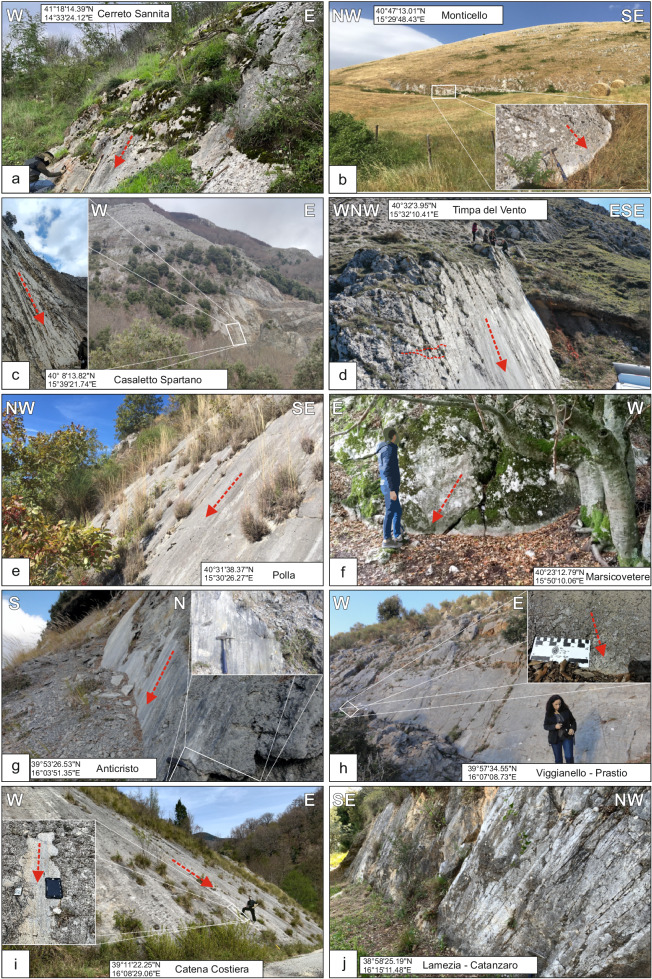
Photographic documentation of some of the fault planes cropping out along the Southern Apennines extensional belt of Italy included in the QUIN 2.0 database. Each photograph has coordinates reported and the slickenlines (red arrows) drawn.

The HFs included in the QUIN 2.0 database^57^ affect different lithologies across the extensional belt. In Campania and Lucania regions, the faults displace platform-to-basinal Meso-Cenozoic limestones, Cenozoic siliciclastic turbidites and Pliocene-Quaternary clastic infill of intra-Apennine extensional basins^66,98,108,111,112^. Conversely, in Calabria, the normal faults either mark the contact between the Pliocene-Quaternary infill of extensional basins and the crystalline basement or displace the crystalline basement^80,113,114^. Representative examples of well-exposed fault planes from different locations of the Southern Apennines are reported in Fig. 3.

The Southern Apennines of Italy have undergone extensive deformation over time, resulting in a presently complex geometric and kinematic setting. The Quaternary extensional belt dissects a pre-existing Mio-Pliocene fold-and-thrust belt (*e.g*.^58,115,116^). The Miocene-Pliocene compressional phase was driven by the convergence of the Eurasia and Nubia plates, involving the opening of the Tyrrhenian Sea and the deformation of the western Adria Plate^59,112,117^. As a result, both the compressional structures and the younger normal faults exhibit an arcuate orientation across the region, ranging from NW-SE in Campania-Lucania to N-S in northern Calabria and NE-SW in southern Calabria and the Peloritani Mountains of Sicily.

Figure 1 shows the alignment of the extensional HF in Southern Italy. Crustal stretching has been suggested to have formed both low-angle (LANFs) and high-angle (HANFs) normal faults^66^. According to some authors^118–120^, the LANFs in the internal (*i.e*., western) side of the extensional belt are associated with an early extensional stage preceding the formation of high-angle normal faults bounding the Quaternary basins. Other authors highlight the contemporaneous activity of east-dipping and antithetic west-dipping faults bounding the major intra-mountains Quaternary basins^66,98,107,121^. The timing of the onset of extensional tectonics also is debated. Some authors propose a regional strike-slip phase during the early stages of intramountain basin development^122–124^, while others argue for dominant extensional tectonics during the same period^66,98^. The age of activity for high-angle extensional tectonics ranges from the Late Pliocene to Early Pleistocene and may become progressively younger toward the east, at a regional scale^3^.

## Data Records

The new FSPs data in QUIN 2.0 are 2,006, while data from the literature are 2,291, for a total of 4,297 FSPs data, grouped in 738 SS distributed quite homogeneously along the intra-Apennines Quaternary faults of the Southern Apennines. Both new and previous FSPs are quality selected (see the “Technical Validation” section) and elaborated to obtain kinematic and deformation axes information. QUIN 2.0 database is stored and available in the Zenodo repository^125^ both in shapefile (.shp) and text (.txt) format.

To make the QUIN 2.0 data tables fully mergeable with QUIN 1.0 and even to promote the future use of integrated databases, we maintained the same structure (Table 1). Therefore, the records are organized into 34 fields, each referring to a single FSP. The 34 fields are organized into three thematic groups (A, B, C) defined as follows: A) FSP identification and SS location (fields 1 to 12); B) FSP geometry, quality ranking and references (fields 13 to 22); C) FSP deformation axes (fields 23 to 34).

**Table 1:** Quin 2.0 thematic groups with examples of records.

Thematic group A: FSP identification and SS location											
Ord_No	SS	FSP	Lon	Lat	Reg	Loc	SS_Pos	Seism	Fault	F_Acronym	F_DipDir
293	MONPAR2	MONPAR2h	15.45300	40.74225	Basilicata	Muro_Lucano	1		Monte_Paratiello	MONPAR	NNE
294	SENER1	SENER1b	15.20040	40.74207	Campania	Senerchia	1	Irpinia_1980_Eq	Senerchia	SENER	E
295	IF8	IF8a	15.31151	40.74028	Campania	Colliano	1	Irpinia_1980_Eq	Irpinia	IF	NE
296	MONTC7	MONTC7a	15.59407	40.73859	Basilicata	Bella	1	Irpinia_1980_Eq	Monticello	MONTC	SSW
297	MONPAR3	MONPAR3a	15.48540	40.73841	Basilicata	Muro_Lucano	1		Monte_Paratiello	MONPAR	NNE
298	MONPAR3	MONPAR3b	15.48540	40.73841	Basilicata	Muro_Lucano	1		Monte_Paratiello	MONPAR	NNE
**Thematic group B: FSP geometry, quality ranking and references**											
**Strike**	**Dip_dir**	**Dip**	**Trend**	**Plunge**	**Rake**	**Kin**	**Q_loc**	**Q_res**	**Ref**		
281.0	11.0	64.0	327.7	56.2	−67.6	LN	A	A	Bel21		
356.0	86.0	57.0	112.1	54.1	−104.9	PN	A	A	Sga20		
315.0	45.0	60.0	45.0	60.0	−90.0	PN	A	A	PP		
141.0	231.0	56.0	190.5	48.4	−64.5	LN	A	A	Bel21		
250.0	340.0	85.0	68.2	19.5	−160.4	RS	B	A	PP		
254.0	344.0	74.0	59.7	40.7	−137.3	RS-N	B	A	PP		
**Thematic group C: FSP deformation axes**											
**P45_Trend**	**P45_Plunge**	**B45_Trend**	**B45_Plunge**	**T45_Trend**	**T45_Plunge**	**P30_Trend**	**P30_Plunge**	**B30_Trend**	**B30_Plunge**	**T30_Trend**	**T30_Plunge**
228.7	63.8	90.7	20.1	354.7	16.1	264.4	69.8	90.7	20.1	360.0	2.0
226.5	73.4	4.3	12.5	96.7	10.8	166.8	76.9	4.3	12.5	273.4	3.8
225.0	75.0	135.0	0.0	45.0	15.0	11.3	90.0	135.0	0.0	45.0	0.0
104.0	67.6	306.0	20.9	213.1	7.7	144.4	68.0	306.0	20.9	38.5	6.3
115.7	17.3	263.7	69.8	22.6	10.1	100.1	19.5	263.7	69.8	8.2	5.3
120.5	41.0	270.6	44.9	16.6	15.4	100.6	44.6	270.6	44.9	5.6	5.0

In addition to the QUIN 2.0 release, we also provide a new release of the HF database with this Data Descriptor. The database is stored in Zenodo^57^ as a shapefile (.shp) and contains 245 linear records (polylines) of the faults hosting the FSPs or neighboring them, traced on a GIS platform. We kept the same structure as the QUIN 1.0 HF table of contents to make the records mergeable. A map view representation of the QUIN 2.0 HF is offered in Fig. 1.

### QUIN 2.0 database records description

In the following, we provide a brief description of each field of the QUIN 2.0 database and of the HF database (in brackets the short names reported in the QUIN’s table):

1. Ordinal Number (Ord_No): uniquely identifies each FSP.
2. Survey Site (SS): contains one or more FSPs; “OF” (Off fault) is reported for those SSs not associated to a certain fault.
3. FSP identification Code (FSP): expressed as the acronym of the fault containing the SS, plus a cardinal number which refers to the Survey Site (SS) and a lowercase letter to identify the individual FSP; “OF” (Off fault) is reported for those FSPs not associated to a certain fault.
4. Longitude (Lon) of the FSP in decimal degrees (WGS84).
5. Latitude (Lat) of the FSP in decimal degrees (WGS84).
6. Region (Reg): administrative region of the data location.
7. Locality (Loc): municipality of the data location.
8. Position (SS_Pos): position and prevailing dip-direction of the SS with respect to the Host Fault (1 = SS located on or near the main fault trace within the damage zone; 2 = SS representing syn-kinematic faults outside the damage zone; 3 = SS consisting of faults observed along antithetic structures; 4 = SS comprising scattered Quaternary fault planes).
9. Coseismic Displacement (Seism): it reports when the SS is located on a fault plane displaced during an instrumentally recorded seismic event (earthquake name and year reported); “NaN” = no information.
10. Host Fault (Fault): name of the Quaternary fault hosting the SS; “OF” (Off fault) is reported if the FSP is not associated to a fault.
11. Host Fault Acronym (F_Acronym): acronym of the fault hosting the SS; “OF” (Off fault) is reported if the FSP is not associated to a fault.
12. Host Fault Dip-Direction (F_DipDir): approximate direction of the Host Fault (expressed with respect to the North); “OF” (Off fault) is reported if the FSP is not associated to a fault.
13. Strike (Strike; in degrees): azimuth angle of a FSP plane with respect to the North.
14. Dip direction (Dip_dir; in degrees): direction of the fault plane dip with respect to the North, expressed with the right-hand rule strike direction.
15. Dip angle (Dip; in degrees): angle of dip of the fault plane measured.
16. Trend (Trend; in degrees): direction of the slip vector.
17. Plunge (Plunge; in degrees): dip of the slip vector.
18. Rake: (Rake) calculated using the Aki-Richard’s annotation^54^.
19. Rake-based kinematics (Kin): fault regimes classification (PN = Pure dip-slip Normal fault; NF = Normal fault; NS = Normal Strike; SN = Strike Normal; SSL = Strike-Slip Left; SSR = Strike-Slip Right; PSS = Pure Strike-Slip). Further information on rake ranges can be found in Andrenacci *et al*.^55^.
20. Location quality ranking (Q_loc): precision in the location with respect to the trace of the Host Fault: A = precise location from available GPS and/or available tabulated data sets, or original field map (expected error in the order of a few meters to some tenths of m); B = approximate location from clear maps and sketch (expected error in the order of some hundreds of meters); C = approximate location from not fully clear maps and sketch (expected error up to 1 km).
21. Data resolution quality ranking (Q_res): precision in reporting the FSP attitude data (slickenside and slickenline) from the original reference source to the database: A = data from tablets and published tabulated dataset or original field booklets; B = FSP attitudes derived from clear published stereoplots projections (expected reproduction error in the order of 1–2°); C = FSP attitudes derived either from not so clear and easily readable published stereoplots projections or from fixed −90° rake for dip-slip faults (expected error up to about 5°).
22. Reference (Ref): source paper used for deriving the SS location (Lat and Lon) and the FSP attitude (strike, dip, dip-angle, trend, and plunge). All remaining data from this database are calculated in this work. Data reference key: Alo13^30^; Ama18^62^; Bel21^28^; Bel22^35^; Bon22^63^; B&S00^64^; Bro09^65^; Bro11^66^; Bro17^67^; Bru16^68^; Cas02b^69^; Cif04^70^; Cif07^71^; DB05^72^; DB06^73^; DG13^74^; Fac11^75^; Fer08^76^; Fer19^77^; Fes03^78^; FW12^26^; Gam21^79^; Ghi79^80^; Imp10^81^; Jac01^36^; Mac14^82^; Mas05^83^; San16^84^; Scu20^17^; Sga20^85^; Tan05^86^; Tan07^87^; Tan15^88^; Tor95^89^; Tri18^90^. Data from this paper are labeled as “PP” (*i.e*., present paper).
23. Trend of the P_45_-axis (P_45__trend; in degrees): trend of the shortening axis.
24. Plunge of the P_45_-axis (P_45__plunge; in degrees): plunge of the shortening axis.
25. Trend of the B_45_-axis (B_45__trend; in degrees): trend of the neutral axis.
26. Plunge of the B_45_-axis (B_45__plunge; in degrees): plunge of the neutral axis.
27. Trend of the T_45_-axis (T_45__trend; in degrees): trend of the extension axis
28. Plunge of the T_45_-axis (T_45__plunge; in degrees): plunge of the extension axis.
29. Trend of the P_30_-axis (P_30__trend; in degrees): trend of the minimum extension axis.
30. Plunge of the P_30_-axis (P_30__plunge; in degrees): plunge of the minimum extension axis.
31. Trend of the B_30_-axis (B_30__trend; in degrees): trend of the neutral axis.
32. Plunge of the B_30_-axis (B_30__plunge; in degrees): plunge of the neutral axis.
33. Trend of the T_30_-axis (T_30__trend; in degrees): trend of the maximum extension axis.
34. Plunge of the T_30_-axis (T_30__plunge; in degrees): plunge of the maximum extension axis.

### Host Faults database records description

1. Ordinal Number (Ord_No): it uniquely identifies each fault trace (numbers increase from north to south).
2. Fault longitude start point (X_start): longitude of the trace start point in decimal degrees (dd.mmmmm; WGS84 reference frame).
3. Fault latitude start point (Y_start): latitude of the trace start point in decimal degrees (dd.mmmmm; WGS84 reference frame).
4. Fault longitude endpoint (X_end): longitude of the trace endpoint in decimal degrees (dd.mmmmm; WGS84 reference frame).
5. Fault latitude endpoint (Y_end): latitude of the trace endpoint in decimal degrees (dd.mmmmm; WGS84 reference frame).
6. Region (Reg): administrative region where the fault is located (in the case of multiple regions, they are all reported from north to south).
7. Host Fault Name (H_F): name of the fault hosting the SS.
8. Host Fault Key (HF_key): gives information on the generic dip-direction of the fault toward the Tyrrhenian Hinterland (east- to north-dipping normal faults in Fig. 1) or the Adriatic-Ionian Foreland (west- to south-dipping normal faults in Fig. 1).
9. Fault System Name (F_S): name of the fault system consisting of a number of along-strike or perpendicular-to-strike interconnected HF; “N_A” is reported for those HFs not included in a fault system.
10. Fault System Onset (F_S_Onset): the age of the oldest recorded displacement and/or of the oldest syn-tectonic deposits among the HFs attributed to the FS; “N_A” is reported for those HFs not included in a fault system.
11. Fault System Seismogenic Activity (F_S_Seism): evidence of seismogenic or potentially seismogenic activity from integrated geological and seismological data; “NaN” = no information.

## Technical Validation

**Fig. 4:**
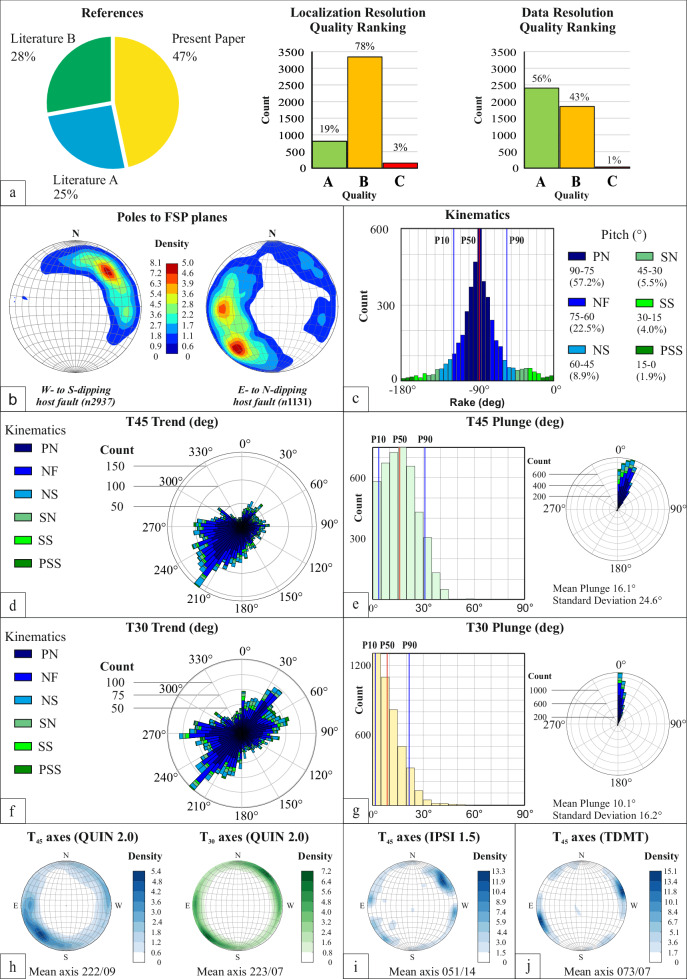
Characteristics of the QUIN 2.0 database. (**a**) Cake diagram describing the percentage of FSP data original or derived from the literature (Literature A = references containing at least one of the authors of the present paper; B = other authors only). The histograms represent the quality rankings for FSP data localization and resolution (A is best). (**b**) Density contours of pole stereographic projections (poles to planes) subdivided for SS lying on Tyrrhenian- and foreland-dipping Host Faults. The poles represented in the stereonet have been grouped on the basis of the Host Faults. To a Host Fault are associated major and minor fault planes falling within its damage zone, and which can therefore have synthetic or antithetic dip-direction with respect to the Host Fault (specified in the “Position” record in the database). (**c**) Rake histogram with rake-based kinematic bins and corresponding pitch angles with relative frequency expressed in percent (key: PN = Pure Normal fault, NF = Normal Fault, NS = Normal Strike-slip fault, SN = Strike-slip Normal fault, SS = Strike-Slip fault, PSS = Pure Strike-Slip fault). (**d**) Trend rose diagram and plunge histogram (**e**) of the tensional deformation axis T_45_, calculated assuming the fracture angle θ = 45°. (**f**) Trend rose diagram and plunge histogram (**g**) of the tensional deformation axis T_30_, calculated assuming the fracture angle θ = 30°. (**h**) T-axis (T_30_ and T_45_) density contours from this database, compared with the (**i**) T-axis density contours of the upper crust (depth <15 km) focal mechanisms in the IPSI’s database and with the (**j**) TDMT database, within the boundaries of the QUIN 2.0 study area.

Of the 4,297 FSPs contained in the QUIN 2.0 release, 47% derive from new observations, while 53% are from the literature (Fig. 4a). The latter are subdivided into literature A (25%) and literature B (28%), which are internal (*i.e*., at least one author of the present paper is author of the paper from that reference) and external (i.e., no authors from this paper are authors of the literature B papers), respectively. As a whole, the new FSPs (47% of the database) and those from the internal literature (28%) represent more than 70% of the database information, giving a general guarantee of homogeneity in the survey methodology and database classification.

Following Lavecchia *et al*.^1^, we assign two different quality rankings defined as Q_loc and Q_res, which represent the resolution of the source information in terms of localization and reproducibility of the FSP, respectively (Fig. 4a). Each quality ranking is subdivided into three classes from A to C, where A represent the most reliable. Regarding Q_loc, ~19% of the QUIN 2.0 FSP data falls into class A, ~78% into class B, and ~3% into class C (A is best). As for Q_Res, ~56% of the data are class A, ~43% class B, and ~1% class C (Fig. 4a).

Still, there are sources of uncertainty in constructing the QUIN databases. These can derive from the original accuracy in the field survey (*e.g*., precision in measuring strikes and dips, trends and plunges), or from imprecisions introduced by digitizing data from pre-existing maps and stereographic projections (*i.e*., operator errors). In the first case, the data acquired with tablet computers or devices equipped with a gyroscope, magnetometer, accelerometer and integrated GPS are the most reliable, especially when checked by acquiring double measurements with a compass. In the second case, the uncertainty may be minimized by considering the quality parameter assigned to the source data (*i.e*., Q_res) and making use of the Andrenacci *et al*.^55^ tool, which highlights, by returning a warning, when the slickenline does not lie exactly on the fault plane.

Approximately 72% of the data is located on HFs dipping toward the Tyrrhenian-hinterland (or on associated antithetic minor structures; west- to south-dipping HF in Figs. 1, 4b), while ~28% is located on HFs dipping toward the Adriatic-Ionian foreland (or on associated antithetic minor structures; east- to north-dipping HF in Figs. 1, 4b). Predominant dip-slip kinematics is evident from the histogram (5° bin) of rake angle values in Fig. 4c. As also observed in the first release, a small percentage of data (~10%) shows a major strike-slip component, with tensional axes coaxial with extensional deformation on average. Figure 4d–g display the orientations (trend and plunge) of the FSP tensional axes (i.e., T_45_ and T_30_) here computed and highlight a near-optimal orientation for most of the FSPs if assuming the Andersonian conditions calculated with θ = 30° (see also Lavecchia *et al*.^1^). Further validation on the FSP comes from comparing these deformation axes with those of the contemporary stress field from focal mechanisms of the IPSI database^7^, and the TDMT^126^. The comparison shows a substantial co-axiality of the Quaternary and present-day deformation field (Figs. 2, 4i,j).

## Usage Notes

Uses of the QUIN database and supporting Host Faults shapefile are fully described in Lavecchia *et al*.^1^. We here recall/highlight some main opportunities:

1. To provide geology-based deformation data and constraints on potentially seismogenic faults in peninsular Italy that lack seismological data due to inactivity in instrumental or historical times, making input data available for seismic hazard assessment.
2. To better identify and characterize seismogenic sources and the stress regime controlling their activity, through comparison of the long-term geological data with present-day geodetic^49,127^ and seismological information^48,110^.
3. To guide geoscientists in the field for detailed studies on active tectonics, seismic hazard, structural geology and historical seismology^18,19,21,31,38,85,128^.
4. To make available a large local-scale dataset that constrains the Quaternary deformation at a regional scale (*i.e*., the whole Apennines), which can be particularly useful in complex areas (for example, the Calabrian Apennines close to the Benioff plane located offshore the Calabrian arc) or in areas known to be seismic gaps such as the Morrone fault system in the Abruzzo region^129^ or the Pollino-Crati fault system in Lucania-Calabria^19,67^.

These objectives may be achieved by exploiting QUIN data with different methods and approaches, including rake-angle interpolation, pseudo-focal mechanism computation^28,51^, stress inversion^19,130^, static-stress interaction analysis^131,132^, and slip-tendency evaluation^133^.

In conclusion, QUIN 1.0 and QUIN 2.0 databases together cover almost the entire extent of peninsular Italy for a length of more than 1,000 km along strike and an area of more than 32,000 km^2^, making available an incredible amount of data (a total of 7,636 FSPs on 1,183 SSs) for future integrations and interpretations by the geological and geophysical scientific community.

## Data Availability

The MATLAB code we used in this work to obtain the FSP deformation axes was developed by Andrenacci *et al*.^55^ for use in Lavecchia *et al*.^1^ and is available from the ZENODO repository (https://zenodo.org/record/5910783)^55^, together with a complete guide to use.
